# Resilience and post‐traumatic growth in the transition to motherhood during the COVID‐19 pandemic: A qualitative exploratory study

**DOI:** 10.1111/scs.13087

**Published:** 2022-05-27

**Authors:** Gill Thomson, Julie Cook, Rebecca Nowland, Warren James Donnellan, Anastasia Topalidou, Leanne Jackson, Vicky Fallon

**Affiliations:** ^1^ Maternal and Infant Nutrition & Nurture Unit (MAINN), School of Community Health and Midwifery University of Central Lancashire (UCLan) Preston UK; ^2^ School of Education, Health and Social Studies Dalarna University Falun Sweden; ^3^ Research Facilitation and Delivery Unit Applied Health Research Hub UCLan Preston UK; ^4^ School of Community Health & Midwifery UCLan Preston UK; ^5^ Department of Psychology University of Liverpool Liverpool UK; ^6^ Research in Childbirth and Health (ReaCH) School of Community Health and Midwifery UCLan Preston UK; ^7^ Institute of Population Health University of Liverpool Liverpool UK

**Keywords:** COVID‐19, growth, perinatal, qualitative, resilience

## Abstract

Most perinatal research relating to COVID‐19 focuses on its negative impact on maternal and parental mental health. Currently, there are limited data on how to optimise positive health during the pandemic. We aimed to bridge this knowledge gap by exploring how women have adapted to becoming a new parent during the pandemic and to identify elements of resilience and growth within their narratives. Mothers of infants under the age of 4 months were recruited as part of a wider UK mixed‐methods study. Semi‐structured interviews with 20 mothers elicited data about how COVID‐19 had influenced their transition to parent a new infant, and if and how they adapted during the pandemic, what strategies they used, and if and how these had been effective. Directed qualitative content analysis was undertaken, and pre‐existing theoretical frameworks of resilience and post‐traumatic growth (PTG) were used to analyse and interpret the data set. The findings show evidence of a range of resilience and PTG concepts experienced during the pandemic in this cohort. Salient resilience themes included personal (active coping, reflective functioning, and meaning‐making), relational (social support, partner relationships, and family relationships), and contextual (health and social connectedness) factors. There was also evidence of PTG in terms of the potential for new work‐related and leisure opportunities, and women developing wider and more meaningful connections with others. Although further research is needed, and with individuals from diverse socioeconomic backgrounds, these findings emphasise the significance of social support and connectivity as vital to positive mental health. Opportunities to increase digital innovations to connect and support new parents should be maximised to buffer the negative impacts of further social distancing and crisis situations.

## INTRODUCTION

Despite the typical psychosocial changes associated with the adjustment to new parenthood, it is well known that potentially ‘*all women can develop mental disorders during pregnancy and in the first year after delivery*’, with a consequent effect on children's growth and development [1]. Major stressful or overwhelming events, emergency, or conflict situations increase the risk of mental disorders in pregnancy and parenthood [1]. The corona virus disease (COVID‐19) has brought unprecedented challenges and changes, with global evidence reporting an increase of mental health problems in some groups [2], including pregnant women and those living with children [3, 4].

Cross‐sectional national surveys undertaken during COVID‐19 have demonstrated a high global prevalence of mental distress in perinatal populations, including depression, anxiety, thoughts of self‐harm, and post‐traumatic stress symptoms [5, 6, 7, 8, 9, 10]. A recent UK study (April–June 2020) was conducted during the first lockdown – a period during which social movements were restricted in the interests of public safety or health. This study highlighted that 43% and 61% of mothers were experiencing clinically relevant depression and anxiety, respectively; alarmingly higher than pre‐pandemic studies using the same measures [11]. These findings are likely attributable to the numerous extraordinary stressors that perinatal women faced during the pandemic – namely reduced social support, restricted birth options, and some maternity care being provided virtually [12, 13, 14, 15]. Most COVID‐19 research relating to maternal and parental mental health has focused on negative impacts [5, 6, 8, 9, 10]. A pathological focus arguably leads to a lack of appreciation of salutogenic factors – those that can promote positive health and well‐being [16]. Currently, there are limited data on positive psychological states during the pandemic [17].

Positive adaptation in the face of adversity can be understood from a resilience perspective. Windle [18] defines resilience as:

The process of negotiating, managing, and adapting to significant sources of stress or trauma. Assets and resources within the individual, their life and environment facilitate this capacity for adaptation and ‘bouncing back’ in the face of adversity (p. 163)

Windle's definition highlights adaptation and ‘bouncing back’ as key features of resilience. Furthermore, although some consider reconfiguration or post‐traumatic growth (PTG) in the face of adversity to be a facet of resilience [19], others consider them to be separate constructs [20]. PTG is defined as both a process and an outcome, as individuals grow and renew following a stressful/traumatic life event, for example, developing more meaningful relationships, spiritual awareness, and appreciation for life [21]. Also integral to Windle's [18] resilience definition is that people do not exist in isolation; they interact with their social and environmental contexts, leading some authors to consider the social ecology of resilience [22]. An ecological approach [23] generally assumes that resilience is a result of the dynamic interplay between internal resources, such as psychological, biological, financial, health, *and* external resources, such as support from private, public, and voluntary services [24]. A parenting‐specific framework that considers resilience from an individual, social, and community perspective has been developed by Young et al [25]. There is some evidence to indicate that resilience is a protective factor against anxiety and stress during the pandemic in pregnant women [26] and in working women with children [27]. However, there have been no previous studies that specifically focus on exploring factors of resilience, and none in relation to PTG, in postpartum women during the COVID‐19 pandemic. We aimed to bridge this knowledge gap.

This article reports on a study undertaken as part of the PRAM (PRegnancy And Motherhood during COVID‐19) project. PRAM aimed to examine the psychosocial experiences of pregnant and postnatal women during different phases of the COVID‐19 pandemic in the United Kingdom [11]. This sub‐study involved semi‐structured interviews to identify how a sample of postnatal women had adapted during the pandemic. The interviews did not specifically ask women about resilience and PTG, rather the methods they had used to maintain positive health. We then drew on theoretical frameworks of resilience [25] and PTG [28] to interpret the data set. This study aimed to provide insights into the resilience and flexibility of human behaviours through a global crisis and aid understanding of ‘what worked’ for these women.

## METHODOLOGY

### Design

A qualitative exploratory design that aimed to capture how women had adapted during the pandemic.

### Participant recruitment and data collection

Participants were recruited as part of the PRAM study. PRAM used online surveys to explore psychosocial experiences of pregnant and postnatal women during social distancing restrictions [11]. Participants were able to indicate at the end of the survey whether they would be willing to take part in an interview to explore their experiences of motherhood with a new infant during COVID‐19. Inclusion criteria for PRAM and the current study included the mother being: 18+ years, English speaking, and residing in the United Kingdom. Respondents also needed to have an infant who was ~4 months of age at the time of the interview. Eligible participants who had indicated that they would be happy to participate in an interview were emailed a separate information sheet and consent form and asked to contact the team if still willing to take part.

Overall, a total of 109 women who participated in the PRAM study expressed an interest in being interviewed. Of these, 61 (56%) responded to the follow‐up invitation; 27 did not meet the inclusion criteria (mainly due to infant age), and 10 did not respond further. Of the 24 who arranged an online interview, four did not attend, leaving 20 participants (17%) who were interviewed for the current study.

At the start of the interview, participants were asked to provide verbal agreement to consent statements read out by the researcher. These data were stored separately to interview data. The interview schedule was created by the co‐authors who have expertise in maternal mental health (GT, VF, LJ), resilience (WD, RN), and growth (GT, WD). Questions involved asking participants about how COVID‐19 had influenced their transition to parenting a new infant and if/what strategies they had used to help them manage the pandemic situation, whether these were new strategies, and if/how they had been effective. It is important to note that we did not ask individuals to make appraisals of resilience or growth; instead, we wanted to capture their experiences of adaptation and change and then explore their accounts for elements of resilience and growth. This approach was undertaken to avoid priming the participants and to acknowledge that individuals may understand or interpret these terms in different ways.

Online (Microsoft Teams) semi‐structured audio interviews were carried out by GT and JC between 22nd July 2020 and 24th September 2020. During this time in the United Kingdom, the ‘eat out to help out’ scheme had been launched for the month of August [29] and socialising was permitted in groups no greater than six, outdoors or indoors [30]. Towards the end of September 2020, further restrictions were reintroduced, including on hospitality opening times, use of face coverings, higher fines for breaking social distancing rules, and stronger lockdown restrictions in some areas [31]. Interviews lasted between 27 and 49 minutes. Data saturation was judged to have occurred when no new concepts were being identified through analysis of newly added transcripts [32]. Participants who agreed were sent a £10 e‐voucher to thank them for their time after the interview had ended.

### Analysis

Audio recordings were transcribed by researchers at the lead author's university. All members of the research team initially read two randomly selected transcripts to get a sense of key issues that were present in the data. This led to more focused reading and discussions within the group to identify theoretical frameworks that would help illuminate the different elements of resilience and growth within the transcripts. The use of theory in qualitative research helps to provide an analytical lens to interpret the data set and to enable a comprehensive conceptual understanding as to why people interact in certain ways [33]. A similar approach has been used in research with other perinatal populations, e.g., to help understand infant feeding behaviours [34, 35].

For this study, a directed qualitative content analysis [36] was used, whereby a pre‐defined coding framework was developed from existing theory that included: a) Young et al.’s parenting resilience framework [25] – a 29‐item matrix that depicts resilience factors in three key areas, ‘personal’, ‘contextual’, and ‘relational’; and b) the five elements of PTG developed by Tedeshi and Calhoun [21, 28], namely appreciation for life, spiritual development, relating to others, personal strength, and new opportunities. A similar approach has been used to apply a theoretical framework to examine resilience among dementia caregivers [37, 38, 39], and the PTG elements have been used to elicit growth within a perinatal population [40, 41]. This analytical process involved developing a categorisation matrix based on the existing frameworks, the theoretical definition of the categories, determining coding rules, and coding the transcripts according to the matrix [36]. Two of the authors (GT, VF) co‐coded two interviews, following which they independently coded the remaining transcripts (*n* = 9 each). Two separate meetings were held to discuss and compare their independent coding for verification purposes – with refinements made as appropriate. All final analytical decisions were revised and agreed upon by all authors.

## RESULTS

Overall, 20 women took part in an interview. Interview participants were aged between 21 and 46 years and infant ages ranged from 5 to 17.5 weeks at the time of the interview. Regarding relationship status, one participant was single, one participant was not living with their partner, and all remaining 18 participants were living with their partner or were married. Nine participants had one child, and 11 participants were multiparous. We also collected the participant's occupations as a proxy for socioeconomic status. One participant was not in paid employment. All other participants were employed (95%), and their occupations were classified using Standard Occupational Categories [42]. Five participants were employed in ‘associated professional occupations’, five in ‘professional occupations’, and nine were employed in ‘managers, directors and senior official’ roles – the highest category of skilled employment (Table 1).

**TABLE 1:** Sociodemographic characteristics of participants

Sociodemographic variables (*n* = 20)	Frequency/range/%
Age – range (mean)	21–46 years (33.7 years)
Infant's age – range (mean)	5w‐17.5w (10.7w)
Marital status (%)	
Married/living with a partner	18 (90.0%)
In relationship/not living with partner	1 (5.0%)
Single	1 (5.0%)
Parity (%)	
One child	9 (45.0%)
Two children	8 (40.0%)
Three children	2 (10.0%)
Four children	1 (5.0%)
Occupation (classified using standard classification levels)^a^	
Level 2 (equates with good general education and typically longer period of work‐related training, e.g., beautician, customer service)	5 (25.0%)
Level 3 (normally requires post‐compulsory education but not normally to degree level, e.g., team manager, chef, freelance writer)	5 (25.05%)
Level 4 (equates to “professional” occupations and high‐level managerial positions, e.g., occupational therapist, chartered accountant, teacher)	9 (45.0%)
Not in paid employment	1 (5.0%)

^a^ Standard Occupational Classification Levels (2020).

In the following sections, we first present the findings mapped against the pre‐defined resilience [25] and PTG [28] theoretical frameworks. It is important to note that participants discussed many different experiences and issues around embarking on motherhood with a new infant under pandemic conditions; there were episodes of poor care, physical and mental ill‐health, and a lack of social support. However, in line with our study aims, we only focused on data related to resilience and growth. This is not to negate or minimise these negative aspects, but with a wider body of literature already dedicated to pathological insights [5, 6, 7, 8, 9, 10, 11], our concern was on identifying factors that could promote positive health. In Table 2, we detail the resilience and PTG factors from the theoretical frameworks that were evident within the narratives, the number of women who referred to these factors, theoretical definitions, and an exemplar quote. We then go on to discuss the more salient concepts (from 50% + of participants' narratives). This approach is advocated within certain methodologies, such as interpretive phenomenological analysis [43], whereby only themes present in at least half the cases are reported. We considered this to be a pragmatic approach that aimed to highlight all the concepts raised while focusing on what was more commonly experienced in relation to resilience and PTG for this group of women.

**TABLE 2:** Resilience Young et al [25] and PTG (Tedeshi & Calhoun, 2004) factors with extracts from interviews

Concept	Definition	Number of participants	Exemplar quote
Resilience factors			
Personal resilience			
Active coping	Taking steps to reduce the negative impact of the stressor	15	*I just make sure I do something every day, at least. Like today probably just silly things like clean my bathrooms (laughing) so today that's my job like ‘cause it's hard with a baby. So yeah, you know, having things to do I suppose has just kept me, you know, going recently*. (P_44)
Reflective functioning	The ability to imagine the mental states of others and through which we understand our own and others' behaviours and responses	14	*I guess kind of like I was saying, I know that we are quite lucky in comparison to some other people. So I've had friends you know that work like on the front‐line and you know they are going to work in quite stressful, scary situations so I understand you know that's really hard and other people – it's affected their jobs and – so I feel like almost making those comparisons I know that I'm quite lucky.* (P_88)
Meaning making	Ability to change, learn, judge meanings and beliefs about the stressor to make it less aversive	10	*And I think with my immediate family like my two children and my husband, we have spent so much time together and I am happy with that because I think wow, I would've never have got that time and those 6 weeks or 7/8 weeks or whatever before [name] was born. I would've never have had that with [toddler name] that one to one time*. (P_45)
Self‐efficacy	Perceived judgement of self‐ability to achieve/attain a specific goal	8	*I'm quite a confident person, I don't take kindly to being brushed off, which sometimes happens, particularly in health care, when you know people are busy, they are under pressure, they want to, get you sorted so they can move on to the next patient quite quickly. I'm quite confident in dealing with that and saying, ‘No, I want information,’* (P_111)
Acceptance	Willingness to tolerate a difficult situation	6	*I think I've coped the best I can with the circumstances and stuff and being able to get my head around the fact that he could potentially get something really dangerous and it be really dangerous for him. But there's nothing I can do to stop, ‘cause I can’t see it’.* (P_77)
Positive self‐concept	Having a positive sense of self	4	*A lot of my friends say I'm quite chilled and quite calm– I do not sort of get anxious*. (P_45)
Intellectual skills	Capacity to understand and make sense of information	4	*I was looking at the Royal College of Obs and Gynae guidelines on Instagram as it was updating towards the end of my pregnancy and when she'd just been born. So, I was looking at them maybe because of my medical background I could access or like understand that kind of guideline to help understand the risks and things like that*. (P_30).
Positive emotion	Expressed positive emotions	2	*Mentally I feel fine because I'm just very sort of happy at being able to have a baby because you know, I was sort of told I wouldn’t be able to have children – so I'm just so happy to have him.* (P_108)
Ask for help	Willingness to engage in help‐seeking behaviours	3	*I was on my own a lot. I could feel my mental health deteriorating and then a couple of weeks ago I really just couldn’t cope anymore and ended up phoning the doctors. They prescribed me sertraline, but I took one tablet and had a really bad reaction to it and so I won’t take it again. But they did give me a referral for psychological therapy, and I had my first session of that today*. (P_41)
Spirituality	Belief in spiritual matters/issues	1	*Yeah, I think belief just our faith like we are both Christians and we have just had to pray every day for God to help us have what we need for that day (laughing) and just going through one day at a time and not looking further ahead than that, but just just relying on friends and family and God.* (P_30)
Contextual resilience			
Health (clinical)	Capacities/opportunities to improve health and well‐being	11	*Yeah – in a way the care they [maternity professionals] were a lot more apologetic* *and attentive and worried that people would be upset and stuff so, I think – yeah. I sort* *of got more sympathetic care do you know what I mean because of the situation we are* *in. So I think I benefitted from it in a way*. (P_108)
Social connectedness	Capacities to make connections with others in our social and personal networks	11	*Yeah so we are part of a church and we have a little a community group thing that was just meeting on Zoom, but people were just messaging us before and bringing us meals and just leaving them at our doorstep and taking her out for a buggy walk and yeah, that that was really good. That was how we kind of got through it really knowing that those people were there (laughing*). (P_30)
Financial health	Having no financial concerns or worries	6	*We have a garden, we have savings. We had access to, to food if we needed it and trying to realise how lucky I am.* (P_110)
Childcare	Accessible childcare provision	5	*We had to form a support bubble with my mum so luckily, we could still have my mum involved, seeing as I've got another child that she had to look after for us. So, I don’t think I would have been able to do it without having that support from my mum*. (P_23)
Community services	Opportunities to access and engage with community‐based assets	2	*We're so fortunate that where we live, we are very rural […] we live near the beach, and as long as I can get out for a little bit to have some fresh air and then I don’t mind having to stay home, that's fine*. (P_16)
Relational resilience			
Social support	Receiving assistance or comfort to help cope with the stressor.	19	*I'm lucky, I've been able to stay in touch with all my friends. We've enjoyed meeting up in the last few weeks and it's strange to be socially distanced to not be able to hug and things, but, there's a depth of relationship with some of my friendships that have weathered many storms and a pandemic is just another one.* (P_110)
Family relationships	Positive relationships with family members	16	*I think we have had time just to sit back and enjoy the fact that we can just have this baby in the family and enjoy time. We've done things in the garden, and the older children are helping to, like, little things, like they are enjoying changing the nappy and taking him in the pram for little walks*. (P_21)
Partner relationships	Positive relationship with partner	12	*I mean, it's just improved. It was, it was a very strong relationship before COVID and before the baby, but it's got even better now*. (P_110)
Access to peer groups	Opportunities to engage with peer group	9	*I’m so relieved that there is something that we can go and do, it’s for 5 weeks, that I can chat to other mums that I don’t know, they are completely new to me. Like there was another little girl that’s the same age as my little boy. And she kicked off all last week. And she loved it this week. And I said, I was like, maybe it’s just because they are that week older and now that they enjoy, or you know, just have a bit of a chat*. (P_94)
Post‐traumatic growth factors			
New possibilities	Stressor has created new opportunities or possibilities that were not present before	11	*So, I suppose thinking about work life balance, I've always done an element of working from home for the past, sort of, I do not know, 10 years or so, with my role. I think my focus going back to the workplace after maternity leave, I do not feel it's necessary in my type of career for me to be in an office environment on a day to day basis. You know, maybe one or 2 days a week yes, but not on a day to day basis. So, I would rather save the 2–3 hours commuting and have that time with my family and I suppose, it's put that into perspective*. (P_56)
Relating to others	The stressor leads to changes in relationships with others such as closer relationships with specific people.	11	*My husband's family are all dotted around the country, so one lives up X* [another region of the country] *way, the other one lives abroad over in X* [Middle‐east country]*. Obviously, there's people all over sort of different areas. So, we do not see each other very often, but through the lockdown we have done Zoom chats every week and that's been really nice ‘cause it's something we never ever did before*. (P_21)
Appreciation for life	The stressor leads to a greater appreciation for life in general	8	*I really value the relationships that I have with family and, you know, not take anything for granted now […]. I feel very grateful as […] previously I had to always be out actually doing something somewhere else, to now be able to potter around the house and enjoying it has been quite nice*. (P_41)
Personal strength	The stressor leads to an increased sense of one's own strength	3	*I think I've kind of have a sense of if you can cope with this, then I can cope with things that are sort of thrown at me, I mean if somebody told me you are going to have 4 months at home being heavily pregnant and then with a newborn with your toddler, with no nursery, without your husband. I would've thought, oh my god, that sounds like hell on earth, like no, I can’t do that but actually because it kind of unfolded and you never knew when it was going to end and it just, the weeks sort of went on and you realised how long you have done. I suppose I would think, you know, actually I can, you can, you are more resilient than you realise.* (P_41)

### Personal resilience

The three most prevalent personal concepts related to *active coping, reflective functioning,* and *meaning‐making*. Fifteen (75%) women referred to using active coping strategies. These included practical strategies such as ‘*keeping busy’*, *‘getting out more*’, and *‘making the most of things [….] like going to the beach and what not* (P_56). Others used emotion‐based strategies designed to alleviate anxieties surrounding the pandemic, such as using a *‘That Was Then, and This Is Now*’ mindfulness‐based strategy to help *‘stay in the moment’* (P_8). Two also used more avoidant coping, by actively avoiding media coverage of government press conferences and variation in infection rates.

*Reflective functioning* was discussed by 14 (70%) women, such as using downward social comparisons to highlight their *‘lucky’* situation; *‘there are a lot of people who are far far far far worse off than we are’* (P_111). Others reflected on difficult decisions they had made during the pandemic: ‘*I think we've done the best that we could've done, I don't regret any of the decisions I've made’* (P_98).

*Meaning‐making* was reported by half (n = 10, 50%) the sample, with women re‐evaluating positive elements of their experiences of social distancing restrictions. Some considered that while restrictions prohibited visitors to their home post‐birth, this also provided a sense of control. One woman reported:

It's stopped too many people coming around. […] we've been able to control that more, so, I didn't feel overwhelmed by visitors. (P_94)

Some women considered how COVID‐19 had brought unexpected positives due to being *‘forced to slow down’* and engage in more family‐centred activities, e.g., ‘*play in the garden all day and that's actually quite nice’* (P_88). Others referred to how the ‘stressor’ had led to positive changes in their self‐beliefs and associated behaviours, e.g., ‘*I was one of those people that I didn’t like to really be at home* [..] *but actually I really enjoyed* [it]’ (P_45).

### Relational resilience

*Social support*, while restricted by social distancing measures, was an important relational factor that promoted resilience for all but one woman (95%) (with specific forms of social support [i.e., partner, family] discussed below). Many women referred to newfound importance of informal social support from friends, neighbours, and colleagues:

You'd maybe have little conversations with different people, but I've genuinely relied on my friend who lives just around the corner and also my colleague at work (P_ 8)

*Family relationships* were discussed by 16 women (80%), generally in the context of valuing spending time with their immediate family because of the lack of distractions from the outside world. Women referred to how the pandemic had provided unique opportunities to spend time together and bond:

I think wow, I would've never have got that time and those six weeks or 7/8 weeks […] one‐to‐one time. (P_45)

Women used existing and new digital technologies to seek support from family members, which often meant they were communicating more regularly:

I've spoken to her [mother] more on the phone than we would've done and Facetimed quite a lot, which we've never done before. (P_8)

Many also considered how enforced face‐to‐face separation provided new perspectives on the meaning of family, stating how *‘privileged’* (P_56) they were to have these relationships and how *‘massively important’* (P_94) they were. The ‘normal’ gatherings that occurred pre‐pandemic also took on new meaning, with women often feeling closer to their families and experiencing a new sense of enjoyment:

We had like a social‐distancing barbeque and we were all so excited (laughing) when normally yeah it's nice having a barbeque with your family […] But actually, that was like so exciting. (P_44)

*Partner relationships* were reported by 12 (60%) women, with social distancing restrictions and furlough schemes allowing many partners to work from home (with ‘normal’ paid paternity leave being 2 weeks). This provided women with practical advantages of additional help: ‘*I've got a spare pair of hands upstairs’* (P_56), and affect‐based benefits in terms of strengthening their relationship, and opportunities for their partners to bond with their new infant:

He's been able to create an incredible bond with her and really get to know her and build up his confidence. (P_8)

### Contextual resilience

Eleven (55%) women discussed *health* and *social connectedness* related issues. Health factors concerned women engaging in activities to improve their physical and mental health: *‘so yeah I now just sort of do lots of exercise, fresh air, relaxation things. They've all helped*’ (P_16). While many women reported negative aspects of COVID‐19 on their maternity care, some referred to how their opportunities for positive health were provided via healthcare professionals:

The midwives were amazing, and the health visitor's been great. Yesterday I went to the GP and had a check and everything, so it's been really great. I've got phone numbers to call if it all starts to go a bit wrong. (P_108)

Some reported how healthcare professionals had provided *‘fabulous’* or *‘more sympathetic care’* (P_108) to ‘*make it the best experience in this circumstance’* (P_98). On one occasion, satisfaction with quality of healthcare was related to the woman and her sister working in a health‐related role: ‘*I have that access to doctors that other people probably don't’* (P_30). Additional procedures to ease women's concerns of infection included safety measures of *‘having a room to myself’* (P_94) or combining hospital appointments (P_23).

*Social connectedness* related to new and/or different ways mothers were connecting with others during the pandemic, via Zoom, Facebook, WhatsApp, email, or Microsoft Teams. Women sought online pregnancy‐ and parenting‐related help and advice, and on some occasions, community members were reported to have adopted an active helping role by taking their infant for a sleep and providing thoughtful acts or gifts, *‘bringing us meals and just leaving them at our doorstep’* (P_30). Sometimes, these were received from unexpected sources:

We're outside numerous times a day, chatting to them [neighbours], they're doing our shopping and if (partner's name) nips out he'll go and sort them out and we've been passing seeds to each other. (P_21)

The practice of ‘clapping the NHS’ on Thursday evenings also provided opportunities for women to meet and form friendships with neighbours and to build a *‘community spirit’* (P_8).

### Growth

*New possibilities* related to developing new interests or paths in life. Eleven (55%) women referred to developing new activities or interests, such as *‘diary writing’*, *‘Pilates’*, growing their own fruit and vegetables, and *‘spending a lot more time outdoors than I would have done’* (P_30). For one woman, the pandemic enabled a change to *‘slow down’* as a new form of *‘existence’* (P_41). Some women spoke of new work directions or patterns, such as a desire to continue home working as ‘*I would rather save the 2‐3 hours commuting and have that time with my family’* (P_56), or to pursue a new career such as counselling or midwifery (P_45).

*Relating to others* was reported by 11 (55%) women; many referred to how COVID‐19 and social distancing had strengthened existing relationships, as well as ‘*widening my social circle’* (P_108). This included forging more meaningful relationships with church members or neighbours who, e.g., ‘*we wouldn't necessarily have like hung out with or spoken to before the lockdown’* (P_30). The shared nature of the pandemic found women valuing *‘friends and families in a different way’* (P_88). Women described *‘closer relationships’* with family members and friends because of the more concerted and repeated efforts to connect during an isolating period: *‘I mean we were always good friends but now we're really, really close friends*’ (P_85).

Others spoke of how the pandemic had created new knowledge about who they could rely on during future stressors. One woman stated:

I think now I know people will rally round each other and people would help me when I need help and support me and our family. (P_30)

Some also considered how the pandemic had enabled them to identify those who *‘really cared’* (P_8) and who *‘you're keeping safe’* (P_44).

## DISCUSSION

The findings show evidence of a range of resilience and PTG concepts reflected in this cohort of women's experiences during the pandemic. Like findings in US‐based studies on coping during COVID‐19 among pregnant and postpartum samples [44, 45], women in the current study used a range of strategies generally centred around promoting self‐care. There was also evidence of positive reflections and making sense of the situation. This included increased time for caregiving and being present with family and an increased sense of control in the early stages of motherhood [44, 45]. Women highlighted the importance of both accessing and benefitting from formal and informal social support to increase resilience. Our findings support the socio‐ecological approach to resilience due to personal, contextual, and relational aspects being identified. We also identified novel evidence of the pandemic creating growth and renewal via new possibilities and more meaningful connections with others.

Although global cross‐sectional studies have shown increased depression, stress, and anxiety during the pandemic [5, 6, 7, 8, 9, 11], it is important to note that these increases were not always dramatic [46]. A national study conducted in the United States on a general population [47] demonstrated evidence of a resilient response to COVID‐19, with early levels of distress diminishing over subsequent months comparable to pre‐pandemic levels. They also found, like the findings in our study, that mindfulness, social support, and meaning‐focused coping predicted better adjustment, reflecting resilience. Other studies have demonstrated individual differences in responses to the pandemic, identifying sub‐groups of resilient individuals in cohorts of pregnant women [48].

Other studies on coping and resilience during the pandemic [44, 45] reinforce the importance expressed by some of our participants of the use of outdoor spaces, such as to talk to neighbours, take exercise, or for ‘socially distant’ events. Several international studies have noted the increased use of and activity in urban and local green spaces during the global pandemic [49], particularly including young families with children [50]. Studies undertaken with a general population found that the intentional use of green spaces has been important for maintaining social interactions during social restrictions [51] and associated with improved physical and mental health and well‐being [52]. However, it was also noted that perceived access to and perceptions of green spaces varies widely between social groups [52] according to existing social vulnerability models [53]. Thus our findings in this regard may be over‐represented in our homogenous sample, with most participants being employed in higher levels of occupational classifications.

Loneliness and social isolation has been reported to increase during the pandemic [54], and one study reported this to be higher in parents than non‐parents [55]. However, women in the current study discussed different ways they received social support. Our findings echo others in that accepting social support from family, friends, and healthcare professionals were important coping strategies [44, 45]. Social support is a well‐known critical buffer for positive mental health (e.g. [56]). Women in the current study highlighted the positivity of increased leisure time, and time with their families, which helped to strengthen friendships and connections. They also spoke of how lockdown measures meant that they were able to spend more uninterrupted time with their immediate family, with positive implications for family bonding and relationships. These findings thereby reflect wider literature about how social connections and leisure can buffer against stress [57, 58], and how social support helps ameliorate the negative impacts of low resilience on participants' mental health [48].

Our findings also demonstrate how the changing use of digital technology during the pandemic has enabled social support and prevented social isolation. This has been reported in other studies. For example, a US‐based study found that digital technology use during the pandemic helped to reduce loneliness more than in‐person contact [59]. Although some evidence has highlighted null effects of online support [60], more nuanced investigations emphasise that it is the quality of interpersonal connections that matter. Women in our study described how the use of online social support was often important because of the positive and meaningful interpersonal connections that were forged with others [61]. These insights emphasise the need to continue and strengthen the use of digital solutions alongside face‐to‐face opportunities, to strengthen friendships and connectivity and to establish strong remote connections to buffer further social distancing and crisis situations.

Our study also found evidence of growth and renewal. Currently, there are only a few studies that explore PTG during the pandemic [62, 63]; although the paucity of studies may relate to growth often occurring after the stressor has passed [21]. In our study, women reported developing new interests and activities and forming new friendships and social connections. Although the pandemic has undoubtedly created high levels of stress, anxiety, and social isolation, our findings highlight how the crisis also enhanced community spirit, provided opportunities to form new and meaningful quality relationships with others, and offered new possibilities and directions for (some) individuals and families.

## STRENGTHS AND LIMITATIONS

This is the first study focussed specifically on positive health through resilience and PTG in women who gave birth during the COVID‐19 pandemic. We drew on robust and relevant theoretical frameworks of resilience and growth to help interpret the findings; furthermore, the use of directed content analysis enabled us to extend elements of these theoretical frameworks and validate their use in a pandemic context.

Although participants were part of the wider PRAM mixed‐methods study, ethical approval for the current sub‐study was obtained separately and did not enable data from the wider study, such as demographics, to be linked to data collected in the current study. Demographic data collected from participants agreeing to take part in the current study were therefore minimised to prevent repetition. The available demographics indicate that the sample is relatively homogenous based on participants' family structures and their occupational groups, with many women having professional roles. This is likely reflective of recruitment methods, with more socially advantaged women being more likely to complete online surveys [11]. Our findings are therefore unlikely to be representative of other cohorts, for example, those with limited access to outdoor/green spaces and/or those with lower socioeconomic status. Although further research to explore resilience and PTG growth in less well‐represented populations (such as disadvantaged, marginalised, and minoritised women) is needed, data collection methods should be carefully considered to increase participation. It is also important to reflect that most of the data were collected during ‘eased’ rather than ‘full’ social distancing requirements and may not be reflective of more restrictive measures being in place.

During the interviews, we did not specifically ask women about resilience and PTG, rather the strategies and methods used to maintain positive health. It is also important to reflect that not all the theoretical domains within the resilience [25] or PTG [28] frameworks were reported. ‘Personal’ related resilience factors not mentioned were: ‘optimism’, ‘flexibility’, and ‘hopefulness’ – potentially because of the ongoing uncertainty of the pandemic situation. ‘Mastery’, ‘secure attachment’, and ‘emotional expressiveness’ are psychological characteristics and beliefs, and non‐reporting of these is perhaps related to the open nature of the questions asked, rather than, e.g., asking individuals to reflect on specific capabilities. Four ‘contextual’‐related resilience factors not mentioned were ‘child temperament’ and ‘gender’, ‘cultural factors’, and ‘social policies’, which could respectively relate to the infant's age at the time of interview, participants' rather homogenous high socioeconomic backgrounds (because of most participants being employed in the top two tiers of occupational classifications), and all participants being subjected to the same social restrictions. ‘Spiritual development’ was the only PTG factor not reported, likely attributed to only one woman disclosing any spiritual related beliefs. Further research could use all the elements of the resilience and growth frameworks [6] as points of discussion, to help elicit how different elements of resilience and growth manifest for different groups of parents.

## CONCLUSION

Notwithstanding the undoubtable and widely documented negative impacts of the COVID‐19 pandemic on new parents, our study adopted a positive psychology focus to uncover how women had adapted during the pandemic and to identify elements of resilience and PTG. Our findings highlight key personal, contextual and relational factors that helped to promote positive health. These include active coping strategies centred around promoting self‐care, and how social distancing provided permission for women to spend uninterrupted time with their infants and families during the transition to parenting a new infant. PTG was also evident through women developing more meaningful connections with others, and opportunities for new careers due to re‐evaluating priorities. Our findings emphasise the significance of social support, and that digital innovations to facilitate positive and meaningful connections should be maximised. Further primary research to ‘test’ the elements of the resilience and growth frameworks should be undertaken, and to identify ‘what works’ in maintaining well‐being in times of adversity for new parents. Overall, the findings of our study add to a growing body of literature that highlights how stress and adversity, on this occasion associated with a global pandemic, also has the potential for new connections, growth, and renewal.

## AUTHOR CONTRIBUTIONS

GT, AT, and VF designed the study, GT provided project management; JC and LJ obtained ethics approval and facilitated recruitment; JC and GT undertook the interviews; GT and VF analysed the data set and prepared the full draft of the paper; WD and RN provided theoretical expertise and input; all authors contributed to writing the manuscript, and all approved the final version.

## ETHICAL APPROVAL

Ethics approval was obtained from the Research Ethics Committee at Liverpool University (No. 7630) and the Health Ethics Panel at the University of Central Lancashire (HEALTH 0082). As it was recognised that the interview had the potential to cause distress, a number of different strategies were included. In the information sheet, participants were advised to not take part in an interview if they would find it too distressing. They were also informed that if they did become distressed, the researcher would suspend the interview and signpost accordingly. Following the interview, participants were sent a debriefing sheet that summarised key issues about the study (e.g., why being undertaken, what would happen to their data) and provided with a list of organisations that could offer emotional‐based support as needed.

## CONFLICT OF INTEREST

None of the authors have any conflicts of interest.
